# Combination of Vancomycin and Cefazolin Lipid Nanoparticles for Overcoming Antibiotic Resistance of MRSA

**DOI:** 10.3390/ma11071245

**Published:** 2018-07-20

**Authors:** Ketki Bhise, Samaresh Sau, Razieh Kebriaei, Seth A. Rice, Kyle C. Stamper, Hashem O. Alsaab, Michael J. Rybak, Arun K. Iyer

**Affiliations:** 1 Use-Inspired Biomaterials & Integrated Nano Delivery (U-BiND) Systems Laboratory, Department of Pharmaceutical Sciences, Wayne State University, Detroit, MI 48201, USA; ketki.bhise@wayne.edu (K.B.); samaresh.sau@wayne.edu (S.S.); hashem.alsaab@wayne.edu (H.O.A.); 2 Anti-Infective Research Laboratory, Department of Pharmacy Practice, Eugene Applebaum College of Pharmacy and Health Sciences, Wayne State University, Detroit, MI 48201, USA; razieh.kebriaei@wayne.edu (R.K.); seth.rice@wayne.edu (S.A.R.); kyle.stamper@wayne.edu (K.C.S.); m.rybak@wayne.edu (M.J.R.); 3 Department of Pharmacy Services, Detroit Medical Center, Detroit, MI 48201, USA; 4 Department of Medicine, Division of Infectious Diseases, School of Medicine, Wayne State University, Detroit, MI 48201, USA; 5 Molecular Imaging Program, Karmanos Cancer Institute, Detroit, MI 48201, USA

**Keywords:** vancomycin, cefazolin, liposomes, nanoparticles, MRSA, macrophage, nephrotoxicity

## Abstract

Vancomycin is the treatment of choice for infections caused by methicillin-resistant *Staphylococcus aureus* (MRSA). Clinically, combinations of vancomycin (VAN) and beta-lactams have been shown to improve patient outcomes compared to VAN alone for the treatment of MRSA bloodstream infections. However, VAN is known to cause nephrotoxicity, which could be ameliorated using biocompatible lipid drug delivery systems or liposomes. Previous attempts have been made for encapsulation of VAN in liposomes; however, drug loading has been poor, mainly because of the high aqueous solubility of VAN. In this study, we report a robust method to achieve high loading of VAN and cefazolin (CFZ) in unilamellar liposomes. Liposomes of sizes between 170–198 nm were prepared by modified reverse phase evaporation method and achieved high loading of 40% and 26% (weight/weight) for VAN and CFZ, respectively. Liposomal VAN reduced minimum inhibitory concentration (MIC) values 2-fold in comparison to commercial VAN. The combination of liposomal VAN (LVAN) and liposomal CFZ (LCFZ) demonstrated a 7.9-fold reduction compared to LVAN alone. Rhodamine dye-loaded liposomes demonstrated superior cellular uptake in macrophage-like RAW 264.7 cells. Fluorescent images of LVAN-encapsulating near-infrared (NIR) dye, S0456 (LVAN-S0456) clearly indicated that LVAN-S0456 had reduced renal excretion with very low fluorescent intensity in the kidneys. It is anticipated that the long circulation and reduced kidney clearance of LVAN-S0456 compared to VAN-S0456 injected in mice can lead to enhanced efficacy against MRSA infections with reduced nephrotoxicity. Overall, our developed formulations of VAN when administered alone or in combination with CFZ, provide a rational approach for combating MRSA infections.

## 1. Introduction

Methicillin-resistant *Staphylococcus aureus* (MRSA) is a world-wide major bacterial pathogen responsible for a wide variety of infections ranging from skin and soft tissue to bone and joint, bloodstream infections, pneumoniae, meningitis, and infective endocarditis. Listed by the Centers for Disease Control and Prevention as a serious level threat to public health, it is responsible for over 80,000 serious infections per year and more than 11,000 deaths. Antibiotics like quinopristin/dalfopristin, telithromycin, linezolid, and daptomycin are licensed for clinical use in treating infections, including those caused by MRSA [1]. While glycopeptides like vancomycin (VAN) remain the preferred treatment of choice for MRSA infections, there are reports suggesting of the emergence of heterogenous VAN-intermediate *S. aureus* (hVISA), and VAN intermediate *S. aureus* (VISA), which render VAN less effective. Combinations of VAN and beta-lactams such as oxacillin and cephalosporin antibiotics have been demonstrated to lower the VAN minimum inhibitory concentration (MIC) against VAN-susceptible, hVISA, and VISA strains [2,3]. More recently, Singh et al., demonstrated that the use of VAN plus cefazolin (CFZ) as a primary therapy against MRSA could prevent the emergence of hVISA or VISA. Of interest, the total daily dose of CFZ required to prevent the emergence of VAN resistance in MRSA was 24-fold lower then typically used to treat infection [4]. Therefore, the use of VAN in combination with CFZ should not only enhance activity against MRSA, but may also prevent the emergence of VAN resistance. In addition, the combination of these agents may reduce the overall VAN exposure, which could lead to a decrease in VAN nephrotoxicity [5].

Nano-formulations like liposomes [6], nanostructured lipid carriers (NLCs), and solid lipid nanoparticles (SLNs) [7], polymeric micelles [8,9,10,11,12], dendrimers [13], have been proven to lower overall drug exposure while increasing the efficacy, as compared to commercially available non-liposomal drugs. Liposomal formulations have become particularly important for drug candidates that have serious adverse side-effects, such as VAN. Entrapment of such drugs protects them from degradation in the body and increases half-life time, thus committing them for targeted action at the site of infection. Liposomal formulations like Doxil™ (Doxorubicin) and AmBisome™ (Amphotericin B) have made successful strides in the clinical setting, reinforcing our belief that liposomal VAN can also be a success story. We believe that VAN liposomes will lower the overall dose exposure of VAN and decrease the associated nephrotoxicity, making it suitable for application in the treatment of MRSA-infected patients. Combination with liposomal CFZ has the potential for lowering the VAN MIC and thereby improving the activity of VAN in the treatment of MRSA infection.

Being highly water-soluble, entrapment of VAN and CFZ in liposomes are reported to be very poor, with drug loading as low as 1% with the thin-film hydration method. To our knowledge, there have been no attempts to increase the drug loading of VAN. We attempted a reverse phase evaporation method to load hydrophilic drugs in liposomes, which resulted in a very high loading, not reported elsewhere. This study explains the formulation of VAN and CFZ liposomes by reverse phase evaporation method and testing for antimicrobial activity against MRSA strains. Since macrophages are known to infiltrate inflamed and infected tissues, we studied the uptake of dye-loaded liposomes *in vitro* in a cultured macrophage-like cell line. Dye-loaded liposomes were also tested for kidney uptake in healthy mice to evaluate their potential impact on nephrotoxicity. Overall, our developed formulations, administered alone or in combination, provide a rational approach for combating MRSA. These formulations can translate into successful animal and patient efficacy with an improvement in patient safety.

## 2. Materials and Methods

### 2.1. Materials

Hydrogenated soybean phosphatidylcholine (HSPC) was purchased from NOF Corporation (Tokyo, Japan). 1,2-distearoyl-sn-glycero-3-phosphoethanolamine with conjugated methoxyl poly(ethylene glycol) (DSPE-mPEG_2000_) was purchased from Nanocs Inc. (Boston, MA, USA). Cholesterol was purchased from Sigma-Aldrich (St. Louis, MO, USA). CFZ was purchased from TCI Chemicals (Tokyo, Japan), Sigma Chemical Company (St. Louis, MO, USA). VAN was purchased from Acros Organics (Geel, Belgium). Sterile phosphate-buffered saline (PBS; purchased from Thermo Fisher Scientific, Waltham, MA, USA) pH 7.4 was used for preparing liposomes. All organic solvents were purchased from Acros Organics and were of 99.5% purity. Bacterial strains MRSA 494 and MSSA ATCC 29213 were obtained from the Anti-Infective Research Laboratory (Detroit, MI, USA). Mueller-Hinton broth (Difco, Detroit, MI, USA) supplemented with 25-mg/L calcium, 12.5-mg/L magnesium was used for all microdilution susceptibility testing and time-kill analyses. Trypticase soy agar (Difco, Detroit, MI, USA) was used for growth and quantification of organisms.

### 2.2. Methods

#### 2.2.1. Preparation of VAN and CFZ Liposomes

Liposomes of VAN and CFZ were prepared by a modified reverse phase evaporation method [14]. For preparation of 1ml 1mM liposomes, the composition of the lipid phase was as follows: HSPC 66.80%, cholesterol 16.46%, and DSPE-mPEG_2000_ 16.72%. Briefly, the lipid phase comprising of HSPC: cholesterol: DSPE-mPEG_2000_ was dissolved in a mixture of diethyl ether: chloroform. The organic phase was evaporated to obtain a thin film of lipid mixture. The resulting thin film was re-dissolved in the same organic phase at the same ratio amounting to 1 mL volume, to which an equal volume of sterile PBS containing drug was added, forming an emulsion. The emulsion was broken by high energy cavitation, followed by evaporation of the organic phase until a clear liposomal solution was obtained. Unencapsulated drugs were separated by filtration (Amicon^®^ Ultra Centrifugal Filters, Millipore Sigma, Burlington, MA, USA; MWCO 3.5 kDa), followed by size extrusion at 60 °C through 200 nm and 100 nm membranes. VAN and CFZ liposomes thus formed were evaluated for their physicochemical and biological characterization.

#### 2.2.2. Chromatography Conditions for Analysis

Sensitive, selective, and robust reverse-phase high performance liquid chromatography (RP-HPLC) methods for detection of VAN and CFZ were developed and validated using Waters^®^ 2695 Separations Module equipped with Waters^®^ 2996 Photodiode Array Detector. Analysis was done by Empower^®^ 3 software connected to the HPLC module. The HPLC analytical methods were validated as per ICH guidelines Q2 (R1) (2005).

Briefly, for VAN, the mobile phase consisted of acetonitrile: water in the ratio 8.5:91.5 (*v*/*v*) with pH adjusted to 3.0 by orthophosphoric acid and supplemented with 0.1% triethylamine to reduce tailing. Chromatographic separations were performed on Waters Resolve™ C18 RP-HPLC column (150 mm × 3.9 mm, 5 μ) at detection wavelength of 230 nm and flow rate isocratic at 1 mL/min. A standard curve was plotted with n = 3. The HPLC analytical (Waters 2695 Separations Module, Waters Corporation, Milford, MA, USA) method for CFZ was developed based on [4] with slight modifications. Briefly, the system consisted of 0.05 M KH_2_PO_4_: acetonitrile (90:10, *v*/*v*) pH 5.0 as the mobile phase at an isocratic flow rate of 1.5 mL/min, and detection wavelength 254 nm. Chromatographic separations were performed on Phenomenex^®^ C8 RP-HPLC column (250 mm × 4.6 mm, 5 μ). Standard curve was plotted with n = 3. For both the drugs, the mobile phase was filtered under a vacuum through a 0.22 μm filter and degassed for 10 min prior to analysis. A 10 μL sample loop was used for analysis.

#### 2.2.3. Evaluation of Percentage Drug Loading and Percentage Encapsulation Efficacy

For this study, the liposomal vesicles were disrupted by addition of methanol and Triton™ X-100 (The Dow Chemical Company, Mitland, MI, USA), and the drug contained within the vesicles was diluted in PBS for quantification by HPLC for VAN and CFZ, respectively. The concentration of drug was intrapolated on VAN and CFZ standard curves and the results were obtained in triplicates. % Drug loading and % encapsulation efficacy were calculated by the following formulae:

$$\%\;\mathrm{Drug}\;\mathrm{loading}\;=\;\frac{\left(\mathrm{Weight}\;\mathrm{of}\;\mathrm{drug}\;\mathrm{contained}\;\mathrm{in}\;\mathrm{the}\;\mathrm{system}\right)}{\left(\mathrm{Total}\;\mathrm{weight}\;\mathrm{of}\;\mathrm{the}\;\mathrm{drug}\;\mathrm{loaded}\;\mathrm{liposomal}\;\mathrm{vesicle}\right)}\times \;100$$

$$\%\;\mathrm{Encapsulation}\;\mathrm{efficacy}\;=\frac{\left(\mathrm{weight}\;\mathrm{of}\;\mathrm{drug}\;\mathrm{contained}\;\mathrm{in}\;\mathrm{the}\;\mathrm{system}\right)}{\left(\mathrm{Total}\;\mathrm{weight}\;\mathrm{of}\;\mathrm{drug}\;\mathrm{loaded}\;\mathrm{in}\;\mathrm{the}\;\mathrm{system}\right)}\times 100$$

#### 2.2.4. Analysis of Vesicle Size by DLS and TEM

Average particle size and polydispersity index of the liposomes were analyzed using dynamic light scattering (DLS, Beckman Coulter Delsa Nano CTM equipped with a 658 nm He-Ne laser, Beckman Coulter, Indianapolis, IN, USA) at ambient temperature. The samples were diluted 100-fold to 1.5 mL with deionized water and the scattered light was detected at an angle of 165°. The average diameter of the vesicles was obtained as a histogram based either on % differential intensity or % cumulative intensity dependent on the average abundance of sizes over 70 scans. The software used for sample analysis was provided by the manufacturer (Beckman Coulter, Delsa Nano ver 2.2, Indianapolis, IN, USA). The morphology of liposomal VAN (LVAN) and liposomal CFZ (LCFZ) was studied using Transmission Electron Microscopy (TEM, H-7500, and Hitach Ltd., Tokyo, Japan). Samples were obtained by placing appropriately diluted nanoparticles onto a carbon-coated 200 mesh copper grid to form a thin film. The film was stained with uranyl acetate, and the excess staining solution was removed with filter paper.

#### 2.2.5. *In Vitro* Release Study

Since both VAN and CFZ are hydrophilic drugs with negative log *P* values (−3.1 and −0.58, respectively), we evaluated the drug release kinetics of CFZ as a representative drug. *In vitro* drug release study was performed by a dialysis method modified in-house. This method used a minimum volume of formulation and release buffer, thus avoiding wastage of materials. Briefly, 200 μL of LCFZ was introduced to Slide-A-Lyzer™ Mini dialysis device, 3.5 kDa MWCO (Thermo Fisher Scientific, Waltham, MA, USA), which was plugged into a 1.5 mL microcentrifuge tube (Thermo Fisher Scientific, Waltham, MA, USA) containing 1 mL of PBS pH 7.4 as release buffer. The assembly was placed on a magnetic stirrer (Thermo Fisher Scientific, Waltham, MA, USA) at 350 rpm, 37 °C. Aliquots of 500 μL were removed from the release buffer at pre-determined time points up to 72 h. An equal volume of release buffer was replaced at every time point to maintain sink-conditions. The aliquots were analyzed by HPLC to quantify CFZ released in the buffer. The graph of % cumulative release versus time (hours) was plotted using GraphPad Prism 7 (GraphPad Software Inc., La Jolla, CA, USA) with n = 2.

#### 2.2.6. Shelf-Life Stability Study

Shelf-life stability of LVAN and LCFZ was assessed by change in vesicle size and polydispersity index after six months using DLS.

#### 2.2.7. Susceptibility Testing

Minimum inhibitory concentrations (MIC) values for VAN and CFZ (commercial drugs and liposomal formulations) both individually and in combination were determined in duplicate by standardized broth microdilution techniques with a starting inoculum of 5 × 10^5^ CFU/mL, according to Clinical and Laboratory Standards Institute guidelines reference. All samples were incubated for 18–24 h at 35 °C.

#### 2.2.8. Time-Kill Analysis

Time-kill assays were performed using liposomal and non-liposomal (commercial) VAN and CFZ, alone and in combination against 494 and 29213 strains using half the MIC for each antibiotic. The starting initial inoculum for the time-kill assays was 10^6^ CFU/mL. Bacterial quantification was determined by sampling aliquots (0.1 mL) at time points of 0, 1, 4, 8, and 24 h. These samples were subsequently serially diluted in cold 0.9% sodium chloride and plated using automatic spiral plating (WASP, DW Scientific, West Yorkshire, UK). After 18–24 h growth on TSA, bacterial colonies were counted using a laser colony counter (ProtoCOL, Synoptics Limited, Frederick, MD, USA). TK curves were generated by plotting mean colony counts (log_10_ CFU/mL) versus time to compare 24 h. The lower limit of detection for the colony counts was 2 log_10_ CFU/mL. To eliminate antibiotic carryover, all samples were diluted sufficiently prior to plating, or if the concentration was close to the MIC post-dilution, they were subjected to additional vacuum filtration with 0.9% sodium chloride. Time-kill curves were constructed by plotting the mean colony counts (log_10_ CFU/mL) versus time. Bactericidal activity was defined as ≥3 log_10_ CFU/mL reduction from baseline. Synergy was defined as ≥2-log_10_ CFU/mL increase in killing at 24 h with the combination, in comparison with the killing by the most active single drug. Combinations that resulted in ≥1-log_10_ CFU/mL bacterial growth in comparison to the least active single agent was considered antagonism. All combinations not meeting the definition of synergy or antagonism were considered indifferent.

#### 2.2.9. Macrophage Uptake Study

RAW 264.7 cells which are macrophage cells derived from tumors induced in male BALB/c mice by the Abelson murine leukemia virus were generously gifted by Shunbin Xu from Wayne State University School of Medicine. 1mM liposomes encapsulating Rhodamine B isothiocyanate (Sigma-Aldrich, St. Louis, MO, USA) were prepared by reverse phase evaporation method with the same lipid phase composition as LVAN and LCFZ. RAW 264.7 cells were sub-cultured in Dulbecco’s Minimum Essential Medium (DMEM) supplemented with 10% Fetal Bovine Serum (FBS) and 1% PenStrep antibiotic mixture. Cells were incubated at 37 °C, 5% CO_2_ in an air-humidified chamber. Cells were plated in petri plates at a population of 1 million cells that achieved up to 80% confluency. The confluent cells were treated with formulation containing 1μM rhodamine B isothiocyanate. Cells were subsequently fixed with 4% formaldehyde solution after analyzing for rhodamine B isothiocyanate signal at 4 h time point and used for staining nuclei with Hoechst 33342 dye (Sigma-Aldrich, St. Louis, MO, USA). For nuclear staining, the fixed cells were treated with 1 μg/mL solution of Hoechst 33342 in PBS and incubated under shaking in the dark at room temperature for 15 min. Cells were examined for Rhodamine B isothiocyanate and Hoechst 33342 signals at 4 h post-treatment under fluorescence microscope with 40× objective (EVOS™ FL Auto Imaging System, Thermo Fisher Scientific, Waltham, MA, USA). The software used for analysis was provided by the manufacturer.

#### 2.2.10. Kidney Uptake in Healthy Mice

All animals used for the kidney uptake studies were performed under the ethical guidelines laid down by the Institutional Animal Care and Use Committee (IACUC) of Wayne State University. To evaluate the potential nephrotoxicity associated with VAN, 1 mM liposomes encapsulating the near infrared (NIR) dye, S0456 were prepared with the same method and composition as described previously. 20 nmol VAN-S0456 liposomes (LVAN-S0456) were injected in athymic nu/nu mice by tail-vein injection. Mice were euthanized 4 h post-injection and imaged under fluorescence microscopy (Bruker Caresteam Xtreme in vivo imaging system, 400W Xenon Illuminator light source) at 750 nm (excitation) and 830 nm (emission) [15].

#### 2.2.11. Statistical Analysis

The statistical analysis was done using GraphPad Prism 7 software (GraphPad Software Inc., La Jolla, CA, USA). The data were expressed as mean ± SD and analyzed using a two-tailed Student *t*-test, or one-way ANOVA followed by a post hoc test. A *p*-value of <0.05 was considered statistically significant.

## 3. Results and Discussion

### 3.1. Chromatography Conditions for Analysis

Drugs like VAN and CFZ absorb light in the ultraviolet range and their determination is likely to be affected by most of the organic compounds. Thus, it was imperative to develop and validate selective and robust HPLC analytical methods for VAN and CFZ for accurate quantification of drug contained within the liposomal vesicles. The HPLC analytical methods for VAN and CFZ were validated as per ICH guidelines Q2 (R1) (2005). The calibration curve for the VAN HPLC method was linear in the range of 2.5 to 50 μg/mL with a correlation coefficient (R^2^) of 0.9991. The LOD and LOQ for VAN were 0.205 μg/mL and 0.684 μg/mL respectively. On the other hand, the calibration curve for CFZ was linear in the range of 1.5625 to 100 μg/mL, with a R^2^ of 1. The LOD and LOQ for CFZ were 0.382 μg/mL and 1.274 μg/mL respectively. Retention times for VAN and CFZ were 6.09 min and 12.42 min respectively.

### 3.2. Evaluation of Percentage Drug Loading and Percentage Encapsulation Efficacy

The % drug loading reflects the total drug that is encapsulated in the liposomes and is a fraction of the total weight of the system. On the other hand, % encapsulation efficacy (or entrapment efficacy) tells us about the amount of drug that is entrapped in the vesicles out of the total drug that is fed into the system. These two related terms are essential in determining the biological efficacy of the formulation and hence, dictate its success.

We achieved high drug loading (weight/weight) with respect to VAN and CFZ in the liposomal vesicles. On extensive optimization of the lipid composition for which the data is not included herewith, we were able to achieve drug loading up to 40% (*w*/*w*) for VAN and up to 26% (*w*/*w*) with respect to CFZ. The % encapsulation efficacies for VAN and CFZ were 75% and 60%, respectively. For both drugs, quantification of drug loading was done based on robust, standardized, and accurate HPLC detection methods.

It is noteworthy to mention here that the % drug loading values were quite a bit higher as compared to liposomes of the same drugs that were earlier reported in literature. The earlier methods employed thin-film hydration method for preparing liposomes in most of the cases. We successfully loaded a high amount of VAN and CFZ in liposomal vesicles by reverse-phase evaporation methods. Thus, it can be safely concluded that this is by far the best method for most hydrophilic drugs.

### 3.3. Analysis of Vesicle Size by DLS and TEM

**Figure 1:**
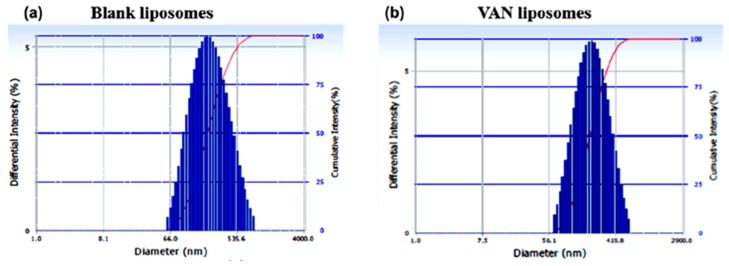

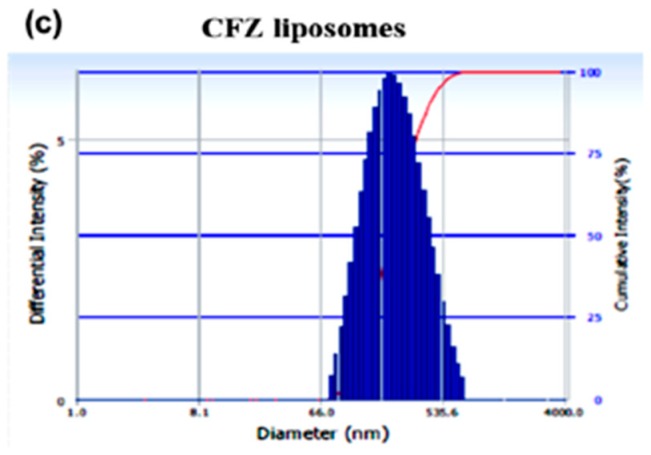
Histograms of vesicle sizes of liposomes (**a**) without drug (blank liposomes); (**b**) vancomycin (VAN) liposomes and (**c**) cefazolin (CFZ) liposomes showing a uniform size distribution.

Liposomes are lipid bilayer vesicles. Hydrophilic drugs like VAN and CFZ are loaded in the inner aqueous compartment of the vesicles. Dynamic Light Scattering (DLS) images for LVAN and LCFZ are shown in Figure 1. LVAN has a mean particle size of 176.5 nm and a polydispersity index (PDI) of 0.201, indicating that a large number of LVAN have a uniform size distribution. Similarly, LCFZ has a mean particle size of 228.4 nm and a PDI of 0.235. Liposomes without the drug (blank liposomes) have a particle size of 210.4 nm and a PDI of 0.268.

**Figure 2:**
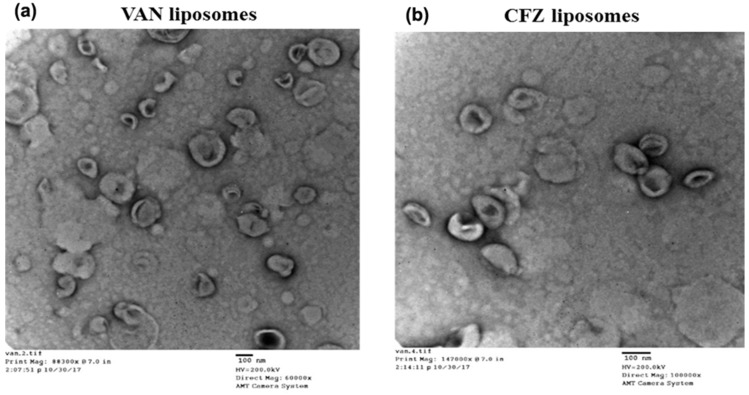
Transmission electron microscopy (TEM) of (**a**) VAN liposomes and (**b**) CFZ liposomes that clearly shows the lipid bilayer formation.

The particle size obtained with DLS was corroborated with that obtained by transmission electron microscopy (TEM). The typical bilayer morphology of liposomes was evident in the TEM images, showing a large aqueous core suitable for encapsulation of highly water-soluble drugs. Figure 2a,b shows the morphology of LVAN and LCFZ respectively.

### 3.4. In Vitro Release Study

**Figure 3:**
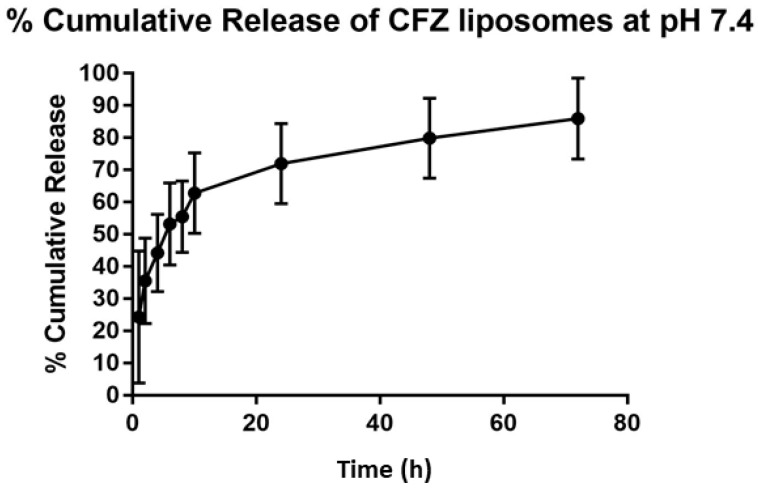
% Cumulative release of CFZ liposomes at pH 7.4.

Since both VAN and CFZ have similar solubility profiles, we evaluated the release profile of LCFZ alone to represent the release kinetics of highly water-soluble drugs. Release kinetics was evaluated at pH 7.4 to mimic intravenous conditions, since CFZ is administered to patients as an injectable dosage form in the clinic. As shown in Figure 3, LCFZ showed immediate release of 20%, followed by a sustained-release pattern over 72 h, with a maximum of 85% release of CFZ. The immediate release acts like a ‘depot’ source of the drug at the site of infection, while the sustained release pattern may ensure maintenance of the drug dose.

### 3.5. Shelf-Life Stability Study

**Figure 4:**
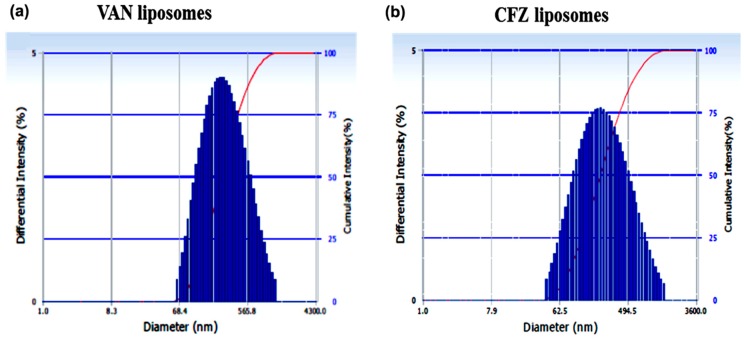
Histograms of vesicle size of (**a**) VAN liposomes and (**b**) CFZ liposomes after six months of shelf-storage.

We determined the shelf-life stability of LVAN and LCFZ over a period of six months by assessing the change in vesicle size and PDI. As demonstrated in Figure 4, the vesicle size of LVAN increased from 176.5 nm to 224 nm with an increase in size distribution as the PDI changes from 0.201 to 0.278. LCFZ did not have a major difference in vesicle size (243 nm, PDI 0.162). For both LVAN and LCFZ, though there were changes in the vesicle size, the integrity of the liposomes seemed to be maintained, since there were no aberrant changes in PDI. The phospholipid and cholesterol components of liposomes were responsible for the integrity and stability of the vesicle over a prolonged period.

### 3.6. Susceptibility Testing

The MIC values for LVAN or LCFZ alone or in combination versus non-liposomal commercial VAN and CFZ are listed in Table 1. The LVAN MIC values were 2-fold lower than that of the commercial product for figure MRSA strain 494. Whereas, LCFZ MICs were >16 fold less than commercial CFZ.

Combination of LVAN-LCFZ demonstrated a 7.9-fold decrease in comparison to LVAN alone while combination of VAN-CFZ showed only a 2-fold reduction in MICs against MRSA strain 494. There was no MIC reduction against ATCC strain 29213 for VAN vs. LVAN; however, LCFZ had a 2-fold reduction vs. CFZ. In addition, there was a 16-fold MIC reduction in with LVAN-LCFZ combination vs. LVAN alone.

### 3.7. Time-Kill Analysis

**Figure 5:**
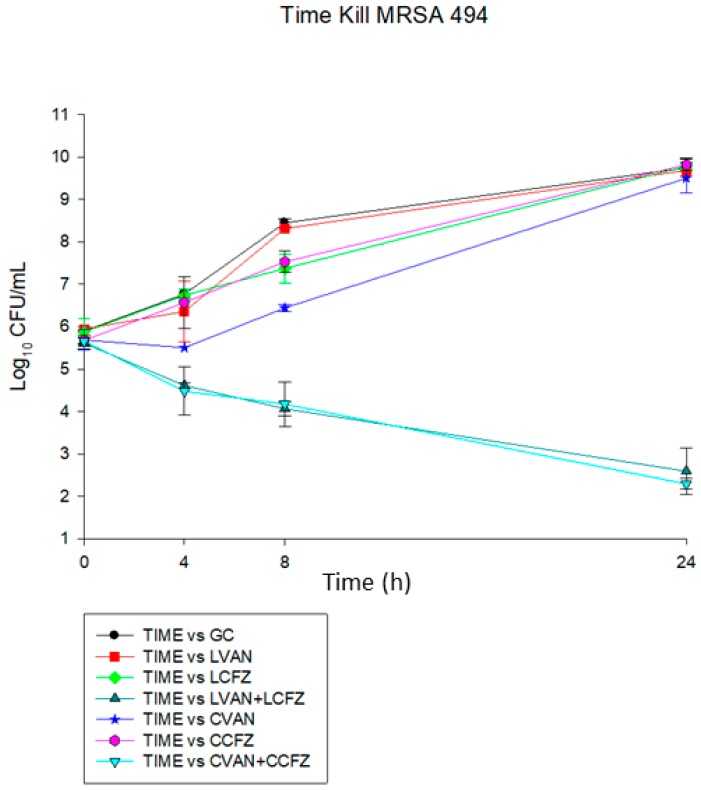
Time kill assay for methicillin-resistant *Staphylococcus aureus* (MRSA) 494 strain.

**Figure 6:**
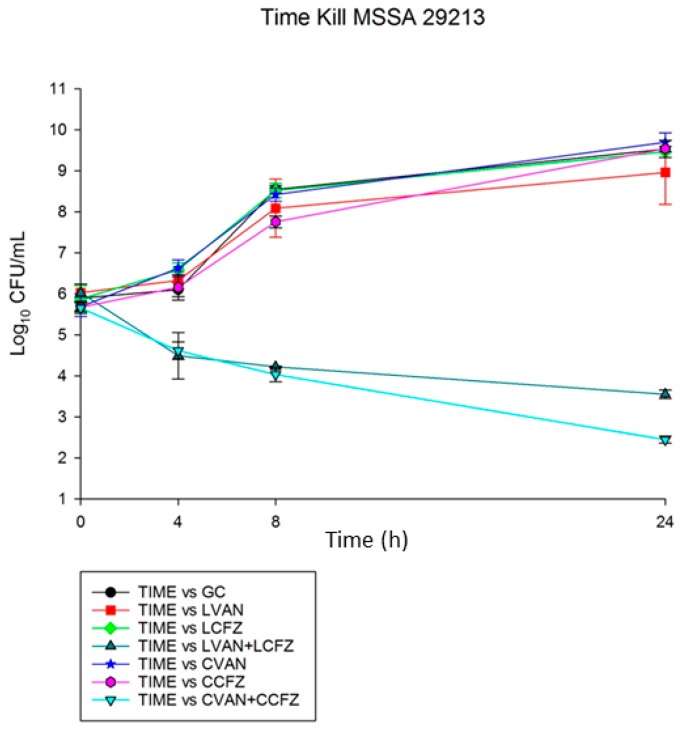
Time kill assay for MSSA 29213 strain.

The combination of both LVAN with LCFZ and commercial VAN with commercial CFZ was synergistic (>2 log CFU/mL) against MRSA 494 and ATCC 29213. This is while single therapies (liposomal or commercial) demonstrated no activity against these organisms. The graphical results are shown in Figure 5 and Figure 6.

### 3.8. Macrophage Uptake Study

**Figure 7:**
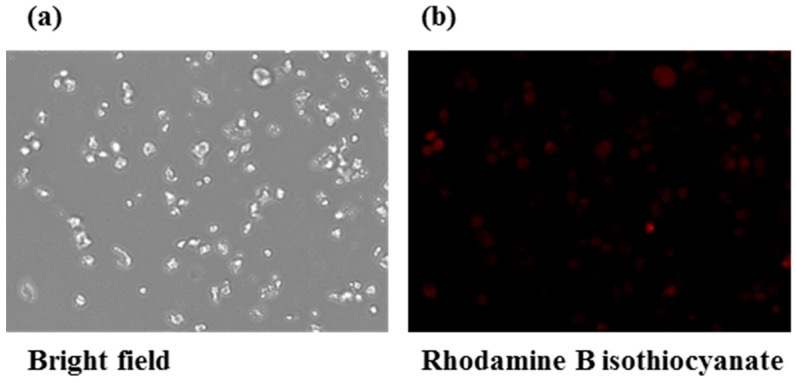

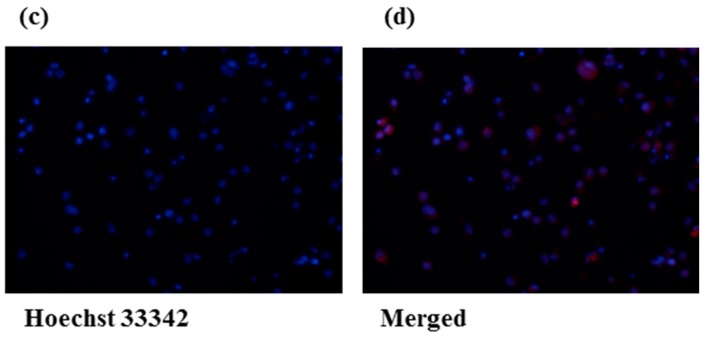
(**a**) Bright field microscopy images of rhodamine B isothiocyanate liposomes treated in RAW 264.7 cells; (**b**) Signal for rhodamine B isothiocyanate channel indicating cytosol staining; (**c**) Signal for Hoechst 33342 channel indicating nuclear staining; (**d**) Merged Rhodamine B isothiocyanate and Hoechst 33342 signals with no co-localization in cells.

One of the mechanisms by which *Staphylococcus aureus* gains antibiotic resistance is by residing in the macrophages, avoiding recognition from the predatory antibiotic molecules. *S. aureus*, upon internalization by macrophages, persist inside the macrophage for 3–4 days, and are fully functional without any signs of apoptosis or necrosis. They are then released into the cytoplasm upon host cell lysis and may thus serve as vehicles for spreading of the infection [16]. Hence, it was thought to be interesting to visualize the penetration of the developed liposomal formulation in cytosol of RAW 264.7 cells, which could be a potential combat for targeting MRSA, and subsequently arresting the spread of MRSA infection. Previous studies have reported uptake of engineered molecules in treatment of MRSA *in vitro* [17,18,19]. Figure 7 shows the fluorescent microscopy images for rhodamine B isothiocyanate-encapsulated liposomes treated in RAW 264.7 cells and observed under rhodamine B isothiocyanate and Hoechst 33,342 filters for cytosol and nuclear staining, respectively. The red signals appeared around the periphery of the blue signals, implying that the Rhodamine B isothiocyanate liposomes had been effectively internalized in the cytosol of cells around the blue-stained nucleus. These results looked promising, considering the developments on reprogramming immune system to combat diseases since the past few years [20,21].

### 3.9. Kidney Uptake in Healthy Mice

**Figure 8:**
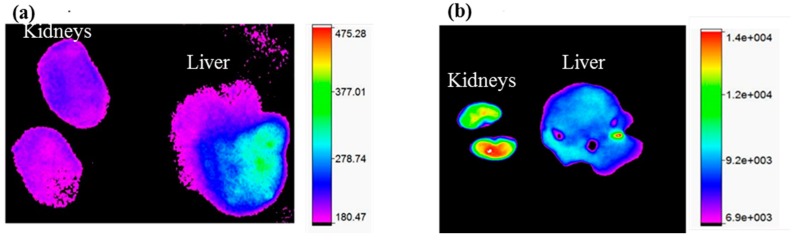
(**a**) shows minimal kidney uptake by LVAN-S0456, suggesting no potential nephrotoxicity for the liposomal formulation; (**b**) shows high kidney uptake for free VAN. Both images were taken after 10 s of exposure at 20 nmol concentration, 4 h post-intravenous administration.

VAN is dose-limiting at higher doses because of associated nephrotoxicity. As shown in Figure 8, fluorescent images of a LVAN-encapsulating NIR dye, S0456 (LVAN-S0456) clearly indicated that LVAN-S0456 delays renal excretion with very low fluorescent intensity and slightly higher accumulation in liver, thus suggesting long circulation of LVAN-S0456 and reduction of nephrotoxicity in MRSA therapy compared to VAN-S0456.

## 4. Conclusions

MRSA remains a superbug worldwide with high morbidity and mortality. Antimicrobial antibiotics are traditionally used in their pure form, but they come with drawbacks associated with dose accumulative toxicity and failure to survive for a long time in biological milieu. Stealth nanoparticles like liposomes provide the drugs with prolonged circulation time and prevent the deposition of the formulation in major organs like heart, kidney, and liver in high concentrations. VAN, the antibiotic used in the present work is well known to cause renal damage. *S. aureus*, which is capable of surviving within the macrophage environment, increases the ability of this organism to spread and contributes to recurrent and relapsing infection. The fact that this organism can survive treatment by residing in the macrophage is one major factor responsible for treatment resistance and the emergence of highly-resistant strains. The formulated liposomes have shown localization in the cytosol of RAW264.7 cells cultured *in vitro.* RAW264.7 are a macrophage cell subtype, and the visualization of cellular uptake strengthens the hypothesis that the formulated liposomes can survive in the macrophage milieu. Studies are underway to evaluate the inhibitory effect of LVAN on *S. aureus* co-cultured with macrophage cells. Future evaluations from our laboratory will evaluate the in vivo pharmacokinetics of these formulations and will further study the impact on nephrotoxicity.

## Figures and Tables

**Table 1:** MIC values of VAN and CFZ (commercial and liposomal) for 494 and 29213 strains.

Strain	MIC Values (mg/L)					
	Commercial VAN	Commercial CFZ	LVAN	LCFZ	LVAN + LCFZ	Commercial VAN + CFZ
494	1	>64	0.5	4	0.063	0.5
29213	0.5	0.5	0.5	0.25	0.031	0.25

## Abbreviations

VAN	Vancomycin
CFZ	Cefazolin
MRSA	Methicillin Resistant *Staphylococcus aureus*
LOD	Limit of Detection
LOQ	Limit of Quantification
LVAN	Liposomal Vancomycin
LCFZ	Liposomal Cefazolin
MIC	Minimum Inhibitory Concentration
RP-HPLC	Reverse Phase-High Performance Liquid Chromatography
DLS	Dynamic Light Scattering
TEM	Transmission Electron Microscopy
PDI	Polydispersity Index
CFU	Colony Forming Units
GC	Growth Control
